# Validation of Using Smartphone Built-In Accelerometers to Estimate the Active Energy Expenditures of Full-Time Manual Wheelchair Users with Spinal Cord Injury

**DOI:** 10.3390/s21041498

**Published:** 2021-02-22

**Authors:** Adrià Marco-Ahulló, Lluïsa Montesinos-Magraner, Luis-Millán Gonzalez, Roberto Llorens, Xurxo Segura-Navarro, Xavier García-Massó

**Affiliations:** 1 Spinal Cord Injury Unit, Physical Medicine and Rehabilitation, University Vall d’Hebron Campus, 08035 Barcelona, Spain; adria.marco@vhir.org (A.M.-A.); lluisa.montesinos@gmail.com (L.M.-M.); xsnavarro@gmail.com (X.S.-N.); 2 Departamento de Educación Física y Deportiva, Universidad de Valencia, 46010 Valencia, Spain; luis.m.gonzalez@uv.es; 3 Neurorehabilitation and Brain Research Group, Instituto de Investigación e Innovación en Bioingeniería, Universitat Politècnica de València, 46022 Valencia, Spain; rllorens@i3b.upv.es; 4 NEURORHB, Servicio de Neurorrehabilitación de Hospitales Vithas, 46007 Valencia, Spain; 5 Departamento de Expresión Musical, Plástica y Corporal, University of Valencia, 46022 Valencia, Spain

**Keywords:** spinal cord injury, smartphone, energy expenditure, physical activity

## Abstract

This study aimed to investigate the validity of using built-in smartphone accelerometers to estimate the active energy expenditures of full-time manual wheelchair users with spinal cord injury (SCI). Twenty participants with complete SCI completed 10 5-min daily activities that involved the upper limbs, during which their oxygen consumption and upper limb activity were registered using a portable gas analyzer and a smartphone (placed on the non-dominant arm), respectively. Time series of 1-min averaged oxygen consumption and 55 accelerometer variables (13 variables for each of the four axes and three additional variables for the correlations between axes) were used to estimate three multiple linear models, using a 10-fold cross-validation method. The results showed that models that included either all variables and models or that only included the linear variables showed comparable performance, with a correlation of 0.72. Slightly worse general performance was demonstrated by the model that only included non-linear variables, although it proved to be more accurate at estimating the energy expenditures (EE) during specific tasks. These results suggest that smartphones could be a promising low-cost alternative to laboratory-grade accelerometers to estimate the energy expenditure of wheelchair users with spinal cord injury during daily activities.

## 1. Introduction

Spinal cord injury (SCI) is defined as damage to the spinal cord, caused by either traumatic causes, such as external forces originating from falls or traffic accidents, or non-traumatic causes, such as inflammation or infections [1,2]. SCI can affect both sensory and motor pathways, as well as the autonomic nervous system, leading to the impairment or loss of sensorimotor function. SCI can result in a lack of physical exercise, which, in turn, can reduce physical fitness and increase the impacts of the injury by increasing the risks of secondary chronic health complications [3,4]. Furthermore, low physical fitness can severely impact the autonomy, quality of life, and self-dependence of individuals who suffer from SCI [5]. In addition to increasing independence, increased physical activity can modulate SCI-induced alterations, assisting with the primary and secondary prevention of various metabolic diseases [6]. Previous literature on SCI has reported that regular physical activity can lead to healthy aging by reducing cardiovascular risks [7], pain [8], and spasticity [9], and improving respiratory function [10]. A previous study performed by Montesinos-Magraner et al. [11] examining individuals with complete SCI found that those individuals who were more physically active had fewer co-morbidities, including fewer diseases and injury-related complications, which supports the recommendation of increased physical exercise in guidelines for this population.

Muscle atrophy below the injury level and a high relative fat mass in individuals with SCI, compared with healthy individuals [12], can result in decreased resting energy expenditures (EEs) among individuals with SCI [13]. Impairments in sensorimotor function can also reduce the activity-associated EE, which describes the energy used by voluntary or involuntary physical movements and mental and emotional processing [14]. Many techniques and instruments have been proposed for the estimation of EE during physical activities [15]. Questionnaires, indirect calorimetry, and heart rate monitors have been used to estimate physical activity levels based on EE. However, questionnaires can be biased, indirect calorimetry is expensive and difficult to apply during daily activities, and heart rate monitors have been shown to have low accuracy; therefore, additional methods must be developed to provide accurate EE estimations of daily activities. 

A systematic review of studies that evaluated commercial monitors, using either default or customized algorithms as well as custom devices and algorithms to assess physical activity, indicated that conventional triaxial accelerometry was the most commonly applied method for estimating EE, and the Actigraph GT3X (ActiGraph, Pensacola, FL, USA) and the GT3X+ appear to be the most widely used accelerometers for EE estimations [16]. The use of accelerometers to quantify EE is based on the premise that EE is determined by the magnitude and rate of muscle forces, which are proportional to accelerations [17]. The same review also identified indirect calorimetry as the gold standard for EE measurements, and the portable Cosmed K4b2 (COSMED, Rome, Italy) was identified as the most commonly used metabolic analyzer for EE measurements. 

The low cost and widespread availability of the current generation of smartphones, which include embedded accelerometers, have resulted in their increased use for the estimation of EE in healthy individuals as an alternative to laboratory-grade devices [18,19]. Although laboratory-grade accelerometers have been validated for the estimation of EE in people with SCI [20,21,22], the validity of using smartphones remains unexplored. A few studies have investigated the validity of using wearable devices such as the Fitbit (Fitbit Inc., San Francisco, CA, USA) [23] or the Apple Watch (Apple Inc., Cupertino, CA, USA) [24,25] to estimate EE in people with SCI or wheelchair users, with promising results. Although these devices can be worn throughout the day, thus allowing for unobtrusive and continuous monitoring of physical activity, their cost might restrict their widespread use. Smartphones, in contrast, have an increasing market penetration, and the number of users has surpassed three billion and is forecast to further grow in the following years [26].

We hypothesized that common smartphones that contain embedded triaxial accelerometers could be used to estimate EE in full-time manual wheelchair users with SCI, representing a low-cost alternative to laboratory-grade instruments. Consequently, this study aimed to validate the use of a mid-range smartphone to estimate EE in a group of full-time manual wheelchair users with SCI, compared with the EE measurements made using a gold standard instrument.

## 2. Methods

### 2.1. Subjects

A convenient, consecutive sample of full-time manual wheelchair users with SCI was recruited from two clinical facilities, Hospital la Fe (Valencia, Spain) and Asociación Provincial de Lesionados Medulares y Grandes Discapacitados (Valencia, Spain). Individuals were considered potential candidates for study participation if they met the following inclusion criteria: (i) had a spinal injury between T2 and L5, diagnosed at least 1 year before enrollment; (ii) were full-time wheelchair users; and (iii) experienced the complete loss of motor function in the lower extremities, as assessed by a score of 0 for the lower extremity items of the American Spinal Injury Association (ASIA) impairment scale. 

Twenty participants, with a mean age of 45.7 (8.37) years, weight of 74.8 (18.05) kg, and height of 173.1 (12.47) cm, enrolled in the study. 

All participants provided written informed consent prior to enrolment in the study. The study was conducted in accordance with the Declaration of Helsinki. The study protocol was approved by the Hospital Universitari Vall d’Hebron Institutional Review Board (PR(ATR)85/2017) on 28 January 2019.

### 2.2. Instrumentation

Accelerometer data were recorded by an MI A2 Android-based smartphone (Xiaomi, Beijing, China), which featured an 8-core Qualcomm Snapdragon 660 and 4 GB of RAM, at a sampling rate of 50 Hz, using a dedicated mobile app, the Physics Toolbox Suite (Vieyra Software, Washington, DC, USA). Indirect calorimetry was measured using the Cosmed K4b2 portable gas analysis system. 

### 2.3. Procedure

**Figure 1:**
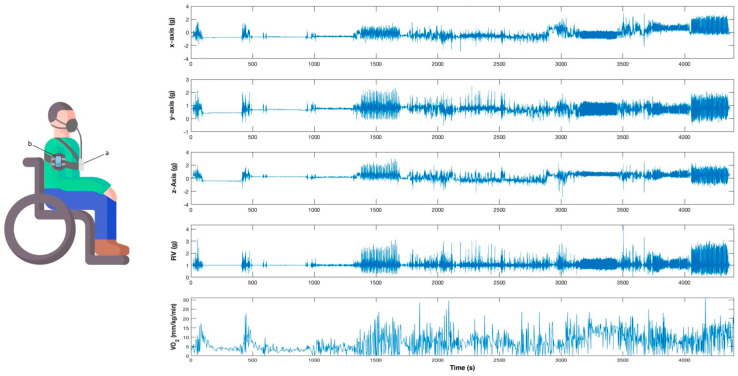
Participant equipped with the instruments (**left**) and an example of the signals acquired by the smartphone and the gas analyzer (**right**). (**a**) Cosmed K4b2. (**b**) Smartphone with built-in accelerometer.

Participants were briefly introduced to the study and were equipped with the gas analyzer and the smartphone, which was fixed to the upper part of the non-dominant arm using a specific smartphone band. The smartphone was positioned on the lateral surface of the arm, midway between the acromion process and lateral epicondyle of the humerus (Figure 1).

All participants completed a routine consisting of 10 5-min activities, with at least 1 min of rest between each activity. The activities were intended to represent different intensities of physical activity for most manual wheelchair users [27,28] (Table 1).

Acceleration and indirect calorimetry were synchronously registered during the testing by using the smartphone and the gas analyzer, respectively (Figure 1). Recording by both instruments was initiated simultaneously to collect data synchronously, and timestamps were set in the Cosmed K4b2 before and after each activity to identify all events.

### 2.4. Data Analysis

A total of 55 variables, including 13 variables for each axis, the resultant vectors, and three variables corresponding to the cross-correlation between the three orthogonal axes, were computed for each activity and were included in the analysis. 

Variables were estimated according to a previous study [21] as follows. Initially, we divided each axis (i.e., x, y, and z) and the resultant vectors into one-minute windows. For each temporal window, we estimated the following: (1) the standard deviation, (2) variance, (3) 10th, (4) 25th, (5) 50th, (6) 75th, and (7) 90th percentiles, and (8) the interquartile range. We also estimated the (9) lag-one correlation of each one-minute time window as a measurement of the temporal dynamics [28]. The acceleration signals were analyzed using a two-level wavelet transform, with the mother wavelet being the Daubechies 2 [29]. We calculated the Euclidean norms of the detail coefficients for the (10) first and (11) second levels of resolution and (12) the approximation coefficients of the second level, commonly referred to as ND1, ND2, and NA2, respectively. We computed the (13) sample entropy for each axis, using a tolerance of 0.3 SD and a pattern length of 2 [30]. Finally, in addition to the 52 variables that resulted from the computation of the 13 variables for each axis and the resulting vector, we estimated the cross-correlation between the three orthogonal axes (x–y, y–z, and x–z cross-correlations) [31] which produced three additional variables, for a total of 55 variables.

We obtained three different multiple linear models: one considering all variables, one considering only linear variables, and one considering only non-linear variables. We used a 10-fold cross-validation method [32] using the average oxygen consumption (VO_2_, mL × kg^−1^ × min^−1^) for every minute as the dependent variable. We used cross-validation, as it is a preventative measure against overfitting [33]. In each fold validation, a training dataset, which included 90% of the total dataset, was used to obtain multiple linear models. A validation dataset that included the remaining 10% of the total dataset was used to determine the goodness of fit for each model. The number of variables that were included in each model (i.e., predictors) was determined using equations containing one to ten estimators for each model. The number of predictors that were included in each model was determined as a tradeoff between performance and computational cost. Finally, the correlation coefficient, the mean squared error, and the mean absolute error (the most widely used variables in the literature [16]) were calculated for each resulting model. Correlations below 0.2 were considered very weak. Correlations ranging from 0.2 to 0.4, 0.4 to 0.6, and 0.6 to 0.8 were considered weak, moderate, and strong, respectively. Finally, correlations greater than 0.8 were considered to be excellent. Signal processing was performed using Matlab R2012a (Mathworks Inc., Natick, MA, USA). 

## 3. Results

**Figure 2:**
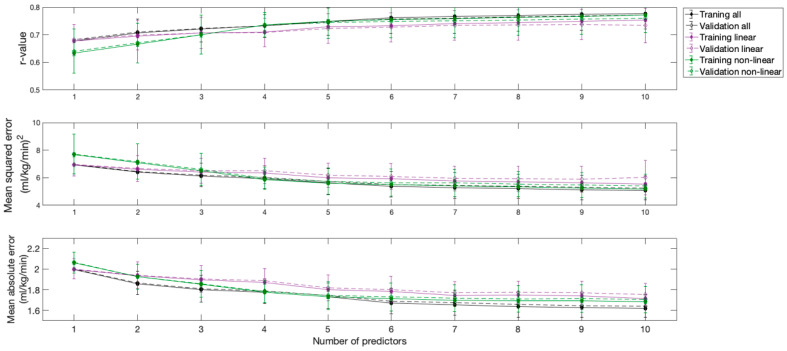
Performance of the models obtained according to the number of predictors used.

The correlation coefficient, the mean square error, and the mean absolute error of the resulting models are shown in Figure 2. All models provided strong correlations. However, the model that included all variables and the model that included only linear variables provided comparable and better results than the model that included only non-linear variables. 

As can be inferred from the inflections of the curves, the first three predictors provided the most substantial contributions to model performance, which were especially evident for the model that considered all variables and the model that considered only linear variables. The inclusion of additional predictors produced minimal changes in the performances of all models. Although considering two predictors in the model that considered only non-linear variables could be sufficient, a third predictor was also considered in this model, to ensure that all models had the same degrees of freedom, facilitating comparisons between them. Therefore, three predictors were considered in all three models, as an acceptable tradeoff between performance and cost.

The applications of the resulting models to the training and validation datasets showed very similar results (Figure 2).

Table 2 shows the equations and performances for all three models. The model that uses all of the variables and the model that only uses linear variables included the same predictors and very similar weights. The model that uses only non-linear variables included different predictors and had a slightly worse performance than the other models.

Finally, Table 3 shows the relative normalized mean squared error and mean absolute error of the three models for each task separately, considering the validation dataset. 

## 4. Discussion

This study assessed the validity of using a smartphone with built-in accelerometers to measure the active EE in full-time manual wheelchair users with SCI during the performance of daily activities. The oxygen consumption and upper limb activities of 20 participants were registered using a portable gas analyzer and a smartphone, respectively, and multiple linear models were estimated based on the linear and non-linear accelerometer variables. Although the performance of all the estimated models was comparable, the best general performance resulted when using either the model that included all of the variables or the model using only the linear variables, and these two models demonstrated a correlation of 0.72, a mean square error of 6.16, and a mean absolute error of 1.76. However, the model that included non-linear variables showed the best performance for some of the investigated activities. These results suggest that smartphones can be used as a potential low-cost alternative to laboratory-grade instruments, which could support their use in both clinical and research activities. 

The data analysis and processing methods used to model predictive algorithms for active EE in this study were similar to those used in a previous study by García-Massó et al. [27], whose performance was highlighted in a recent literature search when searching for other models that used wearable devices to measure activity in people with SCI [34]. The resulting models in the present study demonstrated better performance than previous models, which used data from chest-placed (r = 0.68; mean square error = 10.41; mean absolute error = 2.41) and waist-placed (r = 0.67; mean square error = 10.61; mean absolute error = 2.39) accelerometers [27] and comparable performance to models based on smartwatches placed on the dominant wrist [24]. However, the data registered by accelerometers fixed to the wrist led to models with better performance than those reported in this study, especially when the accelerometer was fixed to the non-dominant wrist (r = 0.86; mean square error = 4.98; mean absolute error = 1.65) [27]. The different performance observed among these studies is likely due to differences in the locations of the instruments. The fixation of the accelerometer to the chest or waist likely limited the ability of these devices to register many of the movements performed during daily activities. Placing the accelerometers on more distal anatomical parts could provide greater sensitivity for the detection of EE during daily activities, such as wheelchair propulsion [20]. In this study, the smartphone was fixed to the upper part of the arm, as its fixation to the wrist would have made the participants uncomfortable and may have diminished the ecological validity of the assessments. Conversely, fixing the smartphone to the participants’ upper arms allowed them to move comfortably and had minimal effects on movement performance. According to previous studies, it could be hypothesized that fixing the smartphone to the chest, the waist, or the wheelchair would have led to worse performance than fixing the smartphone to the arm [27,35]. In contrast, fixing the smartphone to the wrists could have improved the estimation of the EE. Nevertheless, these assumptions should be carefully considered, as the performance of the models could be influenced not only by the position of the accelerometer but also by the number and type of tasks investigated.

The use of laboratory-grade accelerometers could also have improved the estimation of EE, compared with estimations performed using built-in smartphone accelerometers. A previous study examining the sensitivity of laboratory-grade accelerometers placed on the upper arm (similar to the placement used in our study) reported a better performance (r = 0.87) in manual wheelchair users with SCI and other pathologies [20]. The use of smartphone-based accelerometers instead of dedicated accelerometers could explain the reduced accuracy detected in our study, although all of the models built during our study showed strong performance. Moreover, the lower cost and widespread availability of smartphones emphasize the results of our study. Although other studies have examined the use of smartphones with built-in accelerometers to measure EE [36], no previous study has investigated the validation of these devices for manual wheelchair users with SCI. The scant literature that is available on this population has focused on identifying wheelchair movements using the sensor data of a smartphone attached to the wheelchair, and then attempting to extrapolate the level of physical activity based on wheelchair movements [37,38].

The technological advances of the last decade have promoted the widespread use of smartphones, which have become vital parts of people’s lives [39]. The increasing number of sensors being embedded into smartphones and wearable devices has provided researchers with untapped potential for the collection and analysis of pervasive data reliably and at low cost, while being transparent to the study participants. The performance of the models presented in this study for measuring EE in individuals with SCI using commercial smartphones highlights the potential of these devices, and could support their use as wearable and low-cost alternatives to laboratory-grade instruments. The performance of the models built in our study could also endorse the use of smartphones for successive assessments of EE in individuals with complete thoracic SCI, remotely guided by clinicians and researchers, and facilitate the development of mobile health applications that can provide individuals with SCI with accurate estimations of their EE using their own smartphones [36].

Although the range of activities considered in our study was designed to illustrate a representative range of daily activities [27], extrapolations to other activities should be performed with caution. Additionally, the physical characteristics of common smartphones prevent their use in aquatic environments, or while practicing contact sports, such as wheelchair basketball or rugby. Other instruments should be used in these particular situations. However, the performance of the models presented in this study suggests that the estimation of EE in people with complete thoracic SCI is feasible using smartphones, which represent a portable and low-cost alternative to laboratory-grade equipment, and has potential clinical and research implications.

## 5. Conclusions

The present study investigated the validity of using a smartphone to measure the active EE during daily activities in individuals with complete thoracic SCI. Comparable performance was obtained when considering either all of the variables computed from the accelerometer of the smartphone, only the linear variables, or only the non-linear variables. However, the two former options provided slightly better general results. These results suggest that smartphones could be a potential low-cost alternative to laboratory-grade instruments to estimate the EE in individuals with SCI.

## Figures and Tables

**Table 1:** Routine of activities.

Order	Activity	Type	Description
1	Lying down	Sedentary	Participants are required to lie in the lateral decubitus position on a stretcher.
2	Watching TV	Sedentary	Participants are required to sit on their wheelchair and watch TV programs.
3	Working on a computer *	Sedentary	Participants are required to transcribe a text from a news website into a word processing document.
4	Moving items *	Housework	Participants are required to move boxes of different weights (1, 2, and 3 kg) from a shelf on one side of the laboratory to a shelf on the opposite side of the laboratory.
5	Mopping the floor *	Housework	Participants are required to mop the floor of the laboratory at a self-paced speed.
6	Cleaning the windows *	Housework	Participants are required to wipe the windows of the laboratory with a piece of cloth.
7	Ironing *	Housework	Participants are required to iron a set of t-shirts with an iron over an ironing board.
8	Arm-ergometry exercise *	Locomotion	Participants are required to crank an arm ergometer with an intensity that would correspond to a perception of eight points on the OMNI-Res perception scale.
9	Slow propulsion	Locomotion	Participants are required to propel their wheelchair at a comfortable self-selected speed along a long corridor.
10	Fast propulsion	Locomotion	Participants are required to propel their wheelchair at a fast self-selected speed along a long corridor.

* The exercise required real physical objects.

**Table 2:** Description and performance of the resulting models.

Models	Equation	Dataset	Correlation	Mean Square Error (mL·kg^−1^·min^−1^)^2^	Mean Absolute Error (mL·kg^−1^·min^−1^)
All variables	VO^2^ = 3.4921 + 10.784RV_75–25_ − 25.4524Y_VAR_ + 21.0447Y_SD_	Training	0.72	6.08	1.76
		Validation	0.72	6.16	1.76
Linear variables	VO^2^ = 3.4921 + 10.7083RV_75–25_ − 25.4524Y_VAR_ + 21.04487Y_SD_	Training	0.72	6.08	1.76
		Validation	0.72	6.16	1.76
Non-linear variables	VO^2^ = −343.0891 + 503.1303RV_DYN_ + 1.6797RV_ND1_ − 156.1103Y_DYN_	Training	0.71	6.42	1.85
		Validation	0.71	6.48	1.85

RV = resultant vector; Y = *y*-axis; VAR = variance; SD= standard deviation; ND1 = detail coefficient of the first level; 75–25 = interquartile range; DYN = lag-one correlation.

**Table 3:** Relative performance of the resulting models in each task.

	All Variables		Linear Variables		Non-Linear Variables	
	Mean Squared Error	Mean Absolute Error	Mean Squared Error	Mean Absolute Error	Mean Squared Error	Mean Absolute Error
Lying down	9%	23%	9%	23%	9%	24%
Watching TV	11%	26%	11%	26%	11%	30%
Working on a computer	7%	22%	7%	22%	11%	26%
Moving items	6%	19%	6%	19%	8%	21%
Mopping the floor	5%	18%	5%	18%	5%	17%
Cleaning the windows	9%	24%	9%	24%	8%	22%
Ironing	7%	20%	7%	20%	8%	21%
Arm-ergometry exercise	11%	26%	11%	26%	14%	32%
Slow propulsion	10%	27%	10%	27%	9%	25%
Fast propulsion	7%	19%	7%	19%	7%	21%

## Data Availability

The data presented in this study are available on request from the corresponding author.
